# The plant *trans*-Golgi network component ECHIDNA regulates defense, cell death, and endoplasmic reticulum stress

**DOI:** 10.1093/plphys/kiac400

**Published:** 2022-08-26

**Authors:** Lijiang Liu, Li Qin, Luqman Bin Safdar, Chuanji Zhao, Xiaohui Cheng, Meili Xie, Yi Zhang, Feng Gao, Zetao Bai, Junyan Huang, Rishikesh P Bhalerao, Shengyi Liu, Yangdou Wei

**Affiliations:** Key Laboratory of Biology and Genetic Improvement of Oil Crops, Ministry of Agriculture and Rural Affairs, Oil Crops Research Institute, Chinese Academy of Agricultural Sciences, Wuhan 430062, China; Department of Biology, University of Saskatchewan, Saskatoon, S7N 5E2, Canada; Key Laboratory of Biology and Genetic Improvement of Oil Crops, Ministry of Agriculture and Rural Affairs, Oil Crops Research Institute, Chinese Academy of Agricultural Sciences, Wuhan 430062, China; Department of Biology, University of Saskatchewan, Saskatoon, S7N 5E2, Canada; School of Biosciences, University of Nottingham, Leicestershire, LE12 5RD, UK; School of Agriculture, Food and Wine, Waite Research Institute, University of Adelaide, Glen Osmond 5064, Australia; Key Laboratory of Biology and Genetic Improvement of Oil Crops, Ministry of Agriculture and Rural Affairs, Oil Crops Research Institute, Chinese Academy of Agricultural Sciences, Wuhan 430062, China; Key Laboratory of Biology and Genetic Improvement of Oil Crops, Ministry of Agriculture and Rural Affairs, Oil Crops Research Institute, Chinese Academy of Agricultural Sciences, Wuhan 430062, China; Key Laboratory of Biology and Genetic Improvement of Oil Crops, Ministry of Agriculture and Rural Affairs, Oil Crops Research Institute, Chinese Academy of Agricultural Sciences, Wuhan 430062, China; Key Laboratory of Biology and Genetic Improvement of Oil Crops, Ministry of Agriculture and Rural Affairs, Oil Crops Research Institute, Chinese Academy of Agricultural Sciences, Wuhan 430062, China; Key Laboratory of Biology and Genetic Improvement of Oil Crops, Ministry of Agriculture and Rural Affairs, Oil Crops Research Institute, Chinese Academy of Agricultural Sciences, Wuhan 430062, China; Key Laboratory of Biology and Genetic Improvement of Oil Crops, Ministry of Agriculture and Rural Affairs, Oil Crops Research Institute, Chinese Academy of Agricultural Sciences, Wuhan 430062, China; Key Laboratory of Biology and Genetic Improvement of Oil Crops, Ministry of Agriculture and Rural Affairs, Oil Crops Research Institute, Chinese Academy of Agricultural Sciences, Wuhan 430062, China; Department of Forest Genetics and Plant Physiology, Umeå Plant Science Centre, Swedish University of Agricultural Sciences, Umeå, S-901 83, Sweden; Key Laboratory of Biology and Genetic Improvement of Oil Crops, Ministry of Agriculture and Rural Affairs, Oil Crops Research Institute, Chinese Academy of Agricultural Sciences, Wuhan 430062, China; Department of Biology, University of Saskatchewan, Saskatoon, S7N 5E2, Canada; Department of Biology, University of Saskatchewan, Saskatoon, S7N 5E2, Canada

## Abstract

The *trans*-Golgi network (TGN) acts as a central platform for sorting and secreting various cargoes to the cell surface, thus being essential for the full execution of plant immunity. However, the fine-tuned regulation of TGN components in plant defense and stress response has been not fully elucidated. Our study revealed that despite largely compromising penetration resistance, the loss-of-function mutation of the TGN component protein ECHIDNA (ECH) induced enhanced postinvasion resistance to powdery mildew in *Arabidopsis thaliana*. Genetic and transcriptome analyses and hormone profiling demonstrated that ECH loss resulted in salicylic acid (SA) hyperaccumulation via the ISOCHORISMATE SYNTHASE 1 biosynthesis pathway, thereby constitutively activating SA-dependent innate immunity that was largely responsible for the enhanced postinvasion resistance. Furthermore, the *ech* mutant displayed accelerated SA-independent spontaneous cell death and constitutive POWDERY MILDEW RESISTANCE 4-mediated callose depositions. In addition, ECH loss led to a chronically prolonged endoplasmic reticulum stress in the *ech* mutant. These results provide insights into understanding the role of TGN components in the regulation of plant immunity and stress responses.

## Teaser

The *trans*-Golgi network component protein ECHIDNA plays important roles in regulating plant immunity and stress responses.

## Introduction

Plants are exposed to a plethora of fungal, oomycetal, and bacterial pathogens. To cope with those attempted pathogens, plants have evolved diverse defense mechanisms. Plant resistance against filamentous pathogens requires both penetration resistance and postinvasion resistance which are under separate genetic controls (Lipka et al., 2005). Penetration resistance prevents the entry of pathogens into plant body by the deposition of cell wall appositions underneath pathogen-penetration sites (Lipka et al., 2005). Postinvasion resistance terminates the development and/or functioning of the fungal feeding structure such as the intracellular hypha or the haustorium before it extracts enough nutrition from the plant cells for reproduction (Thordal-Christensen, 2003). The regulatory mechanisms of both plant penetration resistance and postinvasion resistance are extremely complex and not fully understood.

The central feature of penetration resistance is the formation of cell wall appositions named papilla, which constitutes a physical and chemical barrier to the invading pathogens (Huckelhoven, 2007). Formation of cell wall appositions is established by site-directed deposition of callose, lignin-like materials, cell wall proteins, reactive oxygen species, and anti-microbial materials at the pathogen-infected sites (Underwood and Somerville, 2008; Meyer et al., 2009). In *Arabidopsis thaliana*, several proteins regulating the penetration resistance are identified with the nonadapted powdery mildew fungus *Blumeria graminis* f. sp. *hordei* (*Bgh*), including the syntaxin PENETRATION 1 (PEN1), the myrosinase PEN2, the ABC transporter PEN3, and the phytochelatin synthase PEN4 (Collins et al., 2003; Lipka et al., 2005; Stein et al., 2006; Meyer et al., 2009; Hématy et al., 2020). PEN1, cooperating with VESICLE-ASSOCIATED MEMBRANE PROTEIN 721 (VAMP721), VAMP722, and SOLUBLE N-ETHYLMALEIMIDE-SENSITIVE FACTOR ADAPTOR PROTEIN 33, allows the fusion of vesicles with the plasma membrane (PM) to transport defense materials to the infection sites, whereas PEN3 transports the antimicrobial chemical products generated by PEN2 and PEN4 across the PM to the apoplastic region. This polarized defense is dependent on effective transport and secretion processes in plant cells. There are two general modes of secretion pathways in eukaryotic cells, that is, the conventional and unconventional secretion pathways, responsible for secretion of various cargoes to the cell surface (Ding et al., 2014). In the conventional secretion pathway (CSP), various cargoes, synthesized in either the cytosol or the rough endoplasmic reticulum (ER), translocate to the ER lumen for processing and subsequently pass through the Golgi and the TGN, where they were further modified, sorted, and packaged before reaching their final cellular destinations. In plants, the CSP is also responsible for the transport of cell wall polysaccharides pectin and hemicellulose, and the cellulose synthases to the cell surface (Anderson and Kieber, 2020). A recent study reported that the TGN components SYNTAXIN OF PLANTS (SYP) 4 group were required for the penetration resistance against powdery mildew fungus by maintaining the transport of VAMP721, extracellular defense proteins, and cell wall-modification enzymes to the infection sites or the apoplast (Uemura et al., 2019), supporting the essential role of CSP components for plant penetration resistance. Nevertheless, the underlying mechanisms are not fully understood.

The penetration resistance is ineffective against host-adapted pathogens as they have evolved the ability to penetrate the host cell wall and produce the feeding structures haustorium (Collins et al., 2003). However, plants can prime postinvasion resistance to stop the growth and development of invaded filamentous pathogens inside the plant body. Typically, the postinvasion resistance is associated with the hypersensitive-response (HR) cell death which is featured by the restricted cell death at the pathogen infection site (Lipka et al., 2005; Wen et al., 2011; Zhang et al., 2015). In Arabidopsis, the postinvasion resistance against the sow thistle powdery mildew strain *Golovinomyces cichoracearum* (*Gc*) UMSG1 or the tobacco powdery mildew strain *Gc* SICAU1 was found to be SA dependent and almost completely overcome in the double mutant *phytoalexin-deficient 4-1 SA induction-deficient 2-1 (pad4-1 sid2-1*) which was defective in salicylic acid (SA) accumulation (Wen et al., 2011; Zhang et al., 2015). The Arabidopsis mutant *powdery mildew resistance 4 (pmr4-1*), losing the ability to deposit callose at the pathogen-infected sites, exhibits SA-dependent enhanced postinvasion resistance to the adapted powdery mildew fungus *Erysiphe cichoracearum* (*Ec*) (Nishimura et al., 2003). Therefore, the HR cell death during the postinvasion resistance against pathogens is closely associated with the SA signaling in plants. Furthermore, SA is reportedly associated with the induction of spontaneous cell death in several lesion-mimic mutants of Arabidopsis including *accelerated cell death 6* (*acd6*), *acd11*, *lesion simulating disease (lsd) 6*, and *lsd7* of which the spontaneous cell death is suppressed by the introduction of exogenic SA hydraoxylase *NahG* (Radojičić et al., 2018). Nevertheless, the spontaneous cell death in the mutants *lsd2 NahG* and *lsd4 NahG* is not eliminated, implying complex regulations of cell death in plants (Radojičić et al., 2018). Notably, the EDS1–PAD4–SAG101 complex-mediated signaling, a key node of plant disease-resistant pathway, is required for the induction of HR cell death during *Bgh* infection in Arabidopsis (Lipka et al., 2005). Presently, the repertoire of plant postinvasion resistance is largely unknown.

As the entry node of CSP, ER is vulnerable to adverse environmental conditions such as heat, cold, drought, or biotic stress which result in accumulations of unfolded or misfolded proteins in the ER lumens and subsequently induce ER stress (Wan and Jiang, 2016; Poor et al., 2019). Upon ER stress, a variety of cellular responses and signal transduction events, referred to as the unfolded protein response, are activated to mitigate ER stress by reducing protein loading over the ER and enhancing ER-associated protein degradation. Failure to do so leads to autophagic or programmed cell death in both animal and plant cells (Wan and Jiang, 2016; Poor et al., 2019). In plants, ER stress can also be induced by mutations in the ER chaperons like BINDING PROTEIN 2 (BIP2; Wang et al., 2005) and ZIP17 (Liu et al., 2007) or other ER-localized proteins such as acetyltransferase NAA50 (Neubauer and Innes, 2020). A recent study reported that loss-of-function of a chloroplast-resident stearoyl-acyl carrier protein desaturase (SUPPRESSOR OF SALICYLIC ACID INSENSITIVITY2) could induce ER stress (Iwata et al., 2018). However, associations of ER stress with the malfunction of other membrane compartments have received less attention in plants.

ECHIDNA (ECH), a conserved and key component of the TGN, is responsible for TGN sorting and secretion functions in Arabidopsis (Gendre et al., 2011). Previous studies have revealed the essential roles of ECH for plant growth and development by maintaining the transport of various cargoes from the TGN to their destinations (Gendre et al., 2011, 2013; Boutte et al., 2013; Fan et al., 2014; McFarlane et al., 2014; Ichino et al., 2020). In this study, we established the functions of ECH in the regulation of plant immunity and stress responses. Although ECH was required for penetration resistance by maintaining the secretion of defense proteins and cell wall components to the cell surface, ECH loss induced enhanced powdery mildew resistance which mainly resulted from the activation of SA-mediated innate immunity due to the ISOCHORISMATE SYNTHASE 1 (ICS1)-derived SA hyperaccumulation. Furthermore, ECH loss resulted in SA-independent spontaneous cell death and constitutive PMR4-mediated callose deposition, as well as accelerated ER stress. Our study provides insights into understanding the role of TGN in the regulation of plant immunity and stress responses.

## Results

### ECH is required for penetration resistance

**Figure 1:**
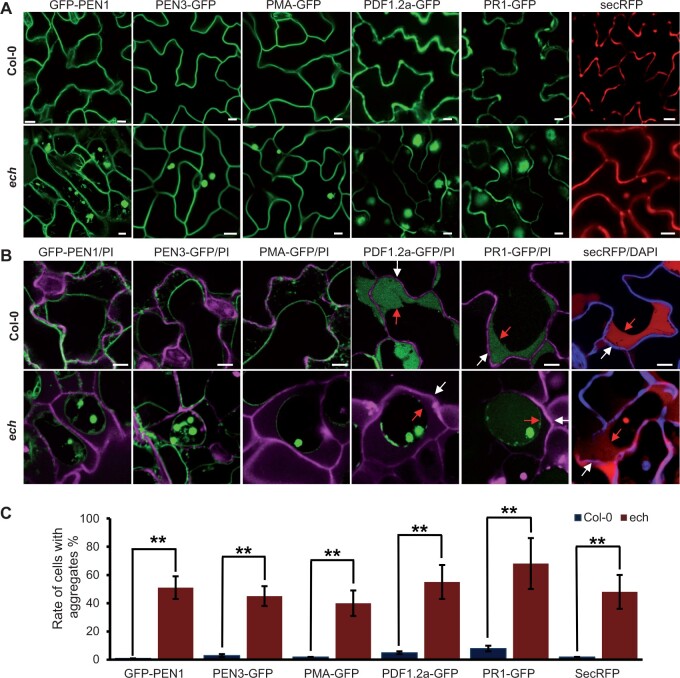
ECH is required for the secretion of penetration resistance-associated proteins. Leaves of the Col-0 or *ech* plants expressing the PM-localized GFP-PEN1 and PEN3-GFP, and the apoplastic defense proteins PDF1.2a-GFP and PR1-GFP were examined under a confocal microscope. The secRFP and PMA-GFP are used as the indicators of the PM secretion route and the apoplastic secretion route, respectively. A, Distribution patterns of PM and apoplastic proteins in the leaf epidermal cells of WT and *ech* plants. PM or apoplastic protein aggregates were frequently observed inside the *ech* cells but not in WT. Bars = 20 μm. B, Plasmolysis analysis of the leaf epidermal cells of the WT and *ech* plants expressing above proteins. The plant cell wall was visualized by the PI staining or the DAPI staining. The separate cell wall and PM were indicated with white and red arrows in the right panels, respectively. Bars = 20 μm. C, Measurement of the leaf epidermal cells containing the intracellular aggregates of above proteins. The data were represented as mean ± sd (*n* = 5, in which more than 200 epidermal cells each were examined). Student’s *t* test, ^**^P < 0.01.

Among the four identified penetration-associated proteins (PEN1–4), PEN1 and PEN3 primarily localize at the PM. Previous studies reported that ECH loss impaired the secretion of only a few PM-targeting proteins (Boutte et al., 2013). To investigate the impact of ECH loss on the PM targeting of PEN1 and PEN3, we collected the *A. thaliana* ecotype Col-0 plants expressing GFP-PEN1 or PEN3-GFP and introduced them into the *ech* mutant (*ECH* loss-of-function mutant) by crossing. Confocal microscopy revealed that GFP-PEN1 and PEN3-GFP displayed typical PM signals in the leaf epidermal cells of the wild-type (WT) plants, which were further confirmed by plasmolysis (Figure 1, A and B). In contrast, GFP-PEN1 and PEN3-GFP signals in *ech* were detected both at the PM and the intracellular aggregates revealed by plasmolysis (Figure 1, A and B). The *ech* mutant was reportedly defective in the secretion of the auxin influx carrier AUX1 to the PM and resulted in the formation of intracellular aggregates inside *ech* cells (Boutte et al., 2013). The observation of GFP-PEN1 and PEN3-GFP aggregates inside the *ech* cells strongly supported that ECH loss compromised the trafficking of PEN1 and PEN3 to the PM. Furthermore, we found that PMA-GFP, a PLASMA MEMBRANE PROTON ATPASE often used as the indicator of PM-localized proteins in plants (Lefebvre et al., 2004), was also retained inside the *ech* cells and formed aggregates (Figure 1, A and B). However, these protein aggregates observed inside the *ech* cells were barely detected in the leaf cells of the WT plants (Figure 1C). Consequently, ECH disruption had a general effect in impairing the PM secretory route.

The extracellular matrices of plant cells are filled with various microbe-hostile proteins and metabolites which, as a part of penetration resistance, constitute a chemical barrier to the invading pathogens (Huckelhoven, 2007; Rocafort et al., 2020). Among them, PLANT DEFENSIN PROTEIN 1.2a (PDF1.2a) and PATHOGENESIS-RELATED PROTEIN 1 (PR1) exhibit a broad-spectrum antimicrobial activity against various phytopathogens (Sels et al., 2008). We collected the Arabidopsis ecotype Col-0 plants expressing PDF1.2a-GFP or PR1-GFP and introduced them into the *ech* mutant by crossing to examine the influence of ECH disruption on their subcellular distributions. As expected, strong GFP signals of the PDF1.2a-GFP and PR1-GFP specifically accumulated in the extracellular spaces of the leaf epidermal cells of WT plants revealed by confocal imaging (Figure 1, A and B). Although the GFP signal but not the RFP signal could be reportedly quenched by the acidic pH of apoplast (Gendre et al., 2011), PDF1.2a-GFP signal driven by the 35S promoter was clearly detected in the apoplast of Arabidopsis in our study and a previous study (Watanabe et al., 2013), possibly due to its hyperaccumulation in the apoplast. In *ech*, however, both PDF1.2a-GFP and PR1-GFP were mainly retained inside the *ech* cells and their extracellular accumulations were below the detection levels, indicating a defect in the apoplastic secretion route in *ech* cells (Figure 1, A and B). We also detected the signal of SECRETED RED FLUORESCENT PROTEIN (secRFP), the indicator of apoplastic secretion route in plants, and found it exclusively accumulated in the apoplasts of the WT cells but deposited in both the intracellular aggregates and the apoplast of the *ech* cells (Figure 1, A and B). These intracellular aggregates of various proteins, however, were barely observed in the cells of WT plants (Figure 1C). The secGFP signals retained inside the *ech* cells were reported to partially colocalize with the ER marker BIP (Gendre et al., 2011), suggesting that these intracellular protein aggregates may partially localize in the ER. From these results, we concluded that ECH loss also had a general effect in impairing the apoplast secretion route in plants.

**Figure 2:**
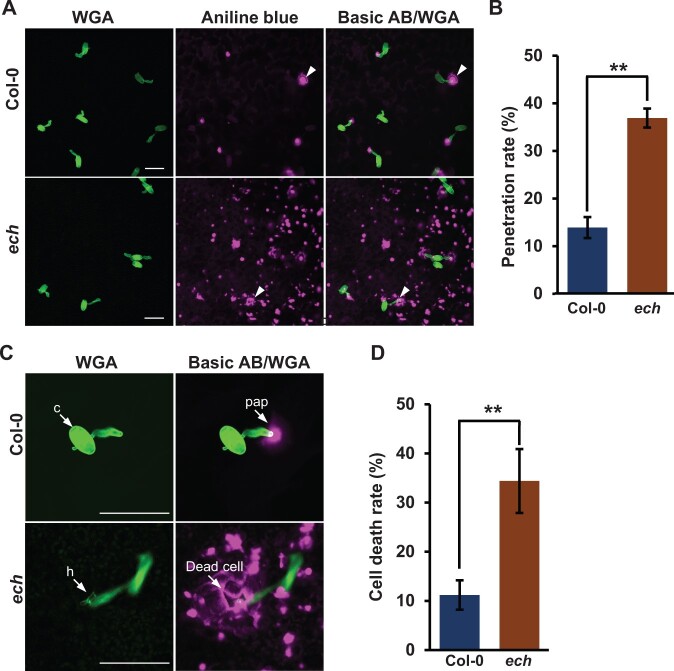
ECH disruption largely compromises the penetration resistance. Four-week-old WT and *ech* plants were inoculated with the conidiospores of nonhost-adapted powdery mildew fungus *Bgh*. A, Germination and infection of *Bgh* on the leaves of the WT and *ech* plants at 36 hpi. Inoculated leaves were subjected to aniline blue (AB) staining for callose and WGA staining for fungal structures. Bars = 50 μm. B, Measurement of the penetration rates of *Bgh* on the leaves of Col-0 and *ech* plants at 36 hpi. Mean ± sd, *n* = 4 in which more than 200 sites each were scored. Student’s *t* test, ^**^P < 0.01. C, Representative infection of *Bgh* on the leaves of the WT and *ech* plants at 36 hpi. Cell death highlighted by intense callosic cell walls occurred after successful *Bgh* penetrations in *ech*. c, conidium; pap, papilla; h, haustorium. Bars = 50 μm. D, Measurement of dead cells induced by *Bgh* infection at 36 hpi. Mean ± sd, *n* = 4 in which more than 200 sites each were scored. Student’s ttest, ^**^P < 0.01.

The general effects of ECH loss in impairing both the PM and apoplast secretion routes imply a compromised penetration resistance in the *ech* mutant. For adapted pathogens like the powdery mildew fungus *Ec* used later in this study, the penetration resistance is ineffective because these pathogens have evolved the ability to penetrate the host cell wall and produce the feeding structure haustorium (Collins et al., 2003). Alternatively, we used the conidia of nonadapted barley powdery mildew fungus *Bgh* to inoculate the leaves of WT and *ech* plants to test the penetration resistance. At 36-h postinoculation (hpi), the fluorescent dye wheat germ agglutinin (WGA) staining revealed that there were no obvious differences between the WT and *ech* mutant in the case of conidiospore germination, germ tube formation, and appressorium development of *Bgh* on the leaves (Figure 2, A and B). In the WT plants, most *Bgh* penetration attempts from mature appressoria were blocked by the preformed callosic papillae and only about 13% of sites showed successful penetrations (Figure 2, A and B). However, the successful penetrations of *Bgh* in *ech* were increased to 38% (Figure 2, A and B), frequently resulting in the production of abnormal bilateral haustoria (Figure 2C). Despite that, HR cell death visualized by intense aniline blue-stained cell walls (Figure 2, C and D) could be activated during the postinvasion of *Bgh* and block its further growth and development inside plant cells (Collins et al., 2003; Lipka et al., 2005). These findings supported that ECH loss largely compromised the penetration resistance and facilitated pathogen infections.

### ECH disruption results in enhanced postinvasion resistance to adapted powdery mildew fungus *Ec*

**Figure 3:**
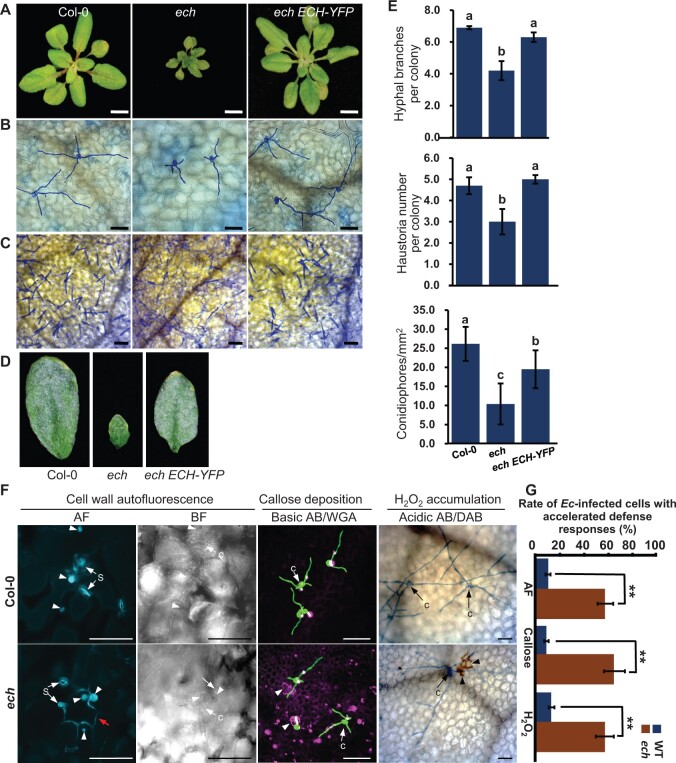
ECH loss induces enhanced postinvasion resistance to *Ec*. A, Phenotypes of 5-week-old plants of WT Col-0, *ech* mutant and complemented line *ech pECH:ECH*-*YFP*. Bars = 1 cm. B and C, hyphae growth and conidiophore production of *Ec* on the leaf surface of above plants at 3 and 11 dpi, respectively. Bars = 100 μm. D, Macroscopic images of the symptoms of *Ec*-infected leaves of indicated lines at 11 dpi. E, Quantitative analysis of the branch and haustorial number of *Ec* per colony at 3 dpi, and conidiophore number of *Ec* per square millimeter at 11 dpi, represented as means ± sd (*n* = 4 in which more than 50 sites each were scored). Different letters indicate statistically significant differences determined by one-way ANOVA with Tukey’s honestly significant difference (HSD) *P* < 0.05. F, Hypersensitive response induced by *Ec* infection in the *ech* mutant. *Ec*-inoculated leaves of Col-0 and *ech* plants were collected at 2 dpi and detected for the cell wall autofluorescence, callose deposition, and H_2_O_2_ accumulation in the *Ec*-infected cells. Fungal structures on the leaf surface were stained with WGA or acidic AB. The penetration sites, autofluorescence or callose-intense cell wall were pointed with arrowheads. AF, autofluorescence; BF, bright field; s, stomata. Bars = 10 μm. G, Quantification of the *Ec*-infected cells with intense autofluorescence, callose-intense cell walls or H_2_O_2_ accumulation. Mean ± sd, *n* = 5 in which more than 50 sites each were scored. Student’s *t* test, ^**^P < 0.01.

As previously reported, ECH loss resulted in a severe dwarf phenotype under normal growth conditions, which could be rescued by expressing *ECH-YFP* in the *ech* mutant (Figure 3A). To further understand the impact of ECH loss on plant disease resistance, we inoculated the WT and *ech* plants with conidiospores of the host-adapted powdery mildew fungus *Ec*. Given a compromised penetration resistance against the nonadapted powdery mildew *Bgh* in *ech*, an increased susceptibility of *ech* plants to *Ec* was expected. However, the *ech* mutant displayed enhanced resistance against *Ec* characterized by lower growth, fewer hyphal branches, and haustoria of *Ec* on the leaves at 5-day postinoculation (dpi), as well as reduced conidiospores and disease symptoms on the leaf surface at 11 dpi compared to the WT plants (Figure 3, B–E).

We next investigated whether HR cell death was associated with the enhanced powdery mildew resistance in *ech*. Pathogen-infected cells undergoing HR cell death are usually accompanied by cell death responses like accumulated autofluorescence and callose, H_2_O_2_ burst, and ion leakage, etc. (Pitsili et al., 2019). We used various tissue-staining techniques to examine whether the cell death responses had occurred in the *Ec*-infected *ech* cells after inoculation with the conidia of *Ec*. At 2 dpi, strong autofluorescence was detected in both the *Ec*-penetration sites and the cell walls of *Ec*-infected leaf epidermal cells of *ech* plants, whereas in the WT plants, this signal was only detected at the *Ec*-penetration sites (Figure 3F). Aniline blue staining revealed that intense callosic cell walls were frequently observed in the *Ec*-infected cells of *ech* plants while in WT plants, the callose deposition was detected only at the *Ec*-penetration sites (Figure 3F). Furthermore, diaminobenzidine tetrahydrochloride (DAB) staining revealed the obvious associations between the H_2_O_2_ accumulation and the *Ec*-infected cells of *ech* plants (Figure 3F). Most *Ec*-infected cells of *ech* plants were accompanied by accelerated cell death responses, which were significantly increased compared to that of WT plants (Figure 3G). These results strongly supported that the enhanced postinvasion resistance associated with the HR cell death was induced during *Ec* infection of the *ech* mutant.

Powdery mildew fungi are typical representatives of biotrophic plant pathogens that feed nutrients from the live cells of plants. In contrast, the necrotrophic plant pathogens like *Sclerotinia sclerotiorum* kill the plant cells before uptaking nutrients from the dead tissues (Liang and Rollins, 2018). We also tested the effect of *ECH* loss on plant resistance against *S. sclerotiorum* using the detached mature leaves. The result showed that the inoculated leaves of *ech* plants developed accelerated disease symptoms and larger death lesions compared to that of the WT plants (Supplemental Figure S1, A and B). Therefore, ECH loss reduced plant resistance to the necrotrophic plant pathogens.

### The enhanced postinvasion resistance to *Ec* is largely dependent on SA-mediated innate immunity

**Figure 4:**
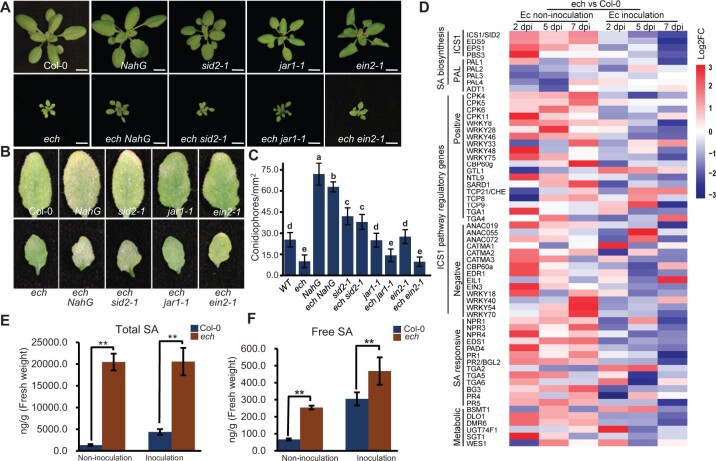
The enhanced postinvasion resistance in the *ech* mutant results from the SA-mediated innate immunity. A, The phenotypes of WT and *ech* plants after introduction of defense phytohormone signaling-defective mutations. Five-week-old plants were photographed. Bars = 1 cm. B, Resistance test of *ech*-associated mutants to *Ec*. Representative leaves of *Ec*-inoculated plants were photographed at 11 dpi. C, Quantitative analysis of *Ec* conidiophore number per square millimeter on the leaves of *Ec*-inoculated plants at 11 dpi in each *ech*-associated mutant. Data are means ± sd (*n* = 6, in which more than 50 sites each were scored). Different letters indicate the statistically significant differences determined by one-way ANOVA with Tukey’s HSD *P* < 0.05. D, Transcriptome profiling of genes involved in SA biosynthesis, regulation, signaling, and metabolization at each indicated time point before or after *Ec* inoculation. Log2FC values were used for the heatmap analysis. E and F, Measurement of total or free SA before or after *Ec* inoculation. Mean ± sd, *n* = 3 in which 15 plants each were performed for SA measurement. Student’s *t* test, ^**^P < 0.01.

To further understand the resistance of *ech* against the powdery mildew disease, we employed a set of mutants or transgenic plants with reduced SA accumulations or defective in jasmonic acid (JA) or ethylene (ET) signaling and crossed them with the *ech* mutant to generate the double mutants. The primers used for screening the double mutants are listed in Supplemental Table S1. Introduction of these mutations or exogenetic genes had no obvious effects on the dwarf phenotype of *ech* plants grown under normal conditions (Figure 4A). Powdery mildew resistance test showed that the leaves of the double mutants *ech NahG* and *ech sid2-1*, in both of which the SA accumulation was dramatically decreased, supported much more conidiospores and developed accelerated disease symptoms than that of *ech* single mutants at 11 dpi (Figure 4, B and C). In contrast, the double mutants *ech jar1-1* (*jasmonate resistant 1-1*) and *ech ein2-1* (*ethylene insensitive 2-1*), in which the JA or ET signaling was impaired, still exhibited strong resistance to *Ec* similar to the level of the *ech* mutant (Figure 4, B and C). These results demonstrated that the enhanced postinvasion resistance of *ech* mutant against *Ec* was mainly regulated by the SA-mediated immunity. We also noted that the production of *Ec* conidiospores on both the leaves of *ech NahG* and *ech sid2-1* plants were slightly below the levels of *NahG* and *sid2-1* plants, respectively, suggesting that other pathways may be activated in the *ech* mutant and play a minor role in regulating the postinvasion resistance against powdery mildew.

To understand how the ECH loss affected SA-mediated immunity, we performed whole-transcriptome shotgun sequencing (RNA-seq). Leaves of the WT and *ech* plants with or without *Ec* inoculation were collected at 2, 5, and 7 dpi for RNA-seq, and more than 60 million raw reads and 50 million clean reads were generated for each sample (Supplemental Table S2). The high quality and correlation index between each repeat indicated the sequencing validity of the RNAseq data (Supplemental Table S3). We collected the identified genes that were involved in SA biosynthesis, signaling, and response pathways and profiled their expressions in the WT and *ech* plants with or without *Ec* inoculation. The result showed that a set of genes involved in the ISOCHORISMATE SYNTHASE 1 (ICS1)-mediated SA biosynthesis pathway were constitutively and coordinately upregulated in *ech* compared to the WT under *Ec*-free conditions (Figure 4D; Supplemental Table S4). In contrast, the genes involved in the PHENYLALANINE AMMONIA-LYASE (PAL)-mediated SA biosynthesis pathway were not obviously altered or even downregulated in *ech* (Figure 4D: Supplemental Table S4). Notably, the transcriptional factors *WRKY*s 8, 28, 48, and 75 that positively regulated the *ICS1* expression were strikingly upregulated in the *ech* mutant under *Ec*-free conditions (Figure 4D; Supplemental Table S4). Furthermore, the SA signaling marker genes *PR1* and *PR2* displayed much higher expressions in *ech* than in WT (Figure 4D; Supplemental Table S4). *Ec* inoculation could dramatically induce the expressions of genes involved in the SA biosynthesis in the WT plants and under *Ec*-inoculated conditions, the expressions of *ICS1*-mediated SA biosynthesis pathway genes were not substantially different between the *ech* and the WT plants, whereas *PR1* expressed much higher in *ech* than in WT plants (Figure 4D; Supplemental Table S4). To confirm whether *ECH* loss led to SA hyperaccumulation, we performed hormone quantification to measure the SA content in *ech* and the WT plants with or without *Ec* inoculations. We found that the total and free SA contents were constitutively hyperaccumulated in *ech* plants under the *Ec*-free condition, 15.3- and 3.8-folds higher than the WT plants, respectively (Figure 4, E and F). When *Ec* challenged, the accumulated total and free SA in *ech* plants were still much higher than in the WT plants, although their productions were induced by *Ec* infection in the WT plants (Figure 4, E and F). In conclusion, ECH loss induced SA hyperaccumulation via ICS1-dependent biosynthesis pathway and subsequent SA-mediated innate immunity.

**Figure 5:**
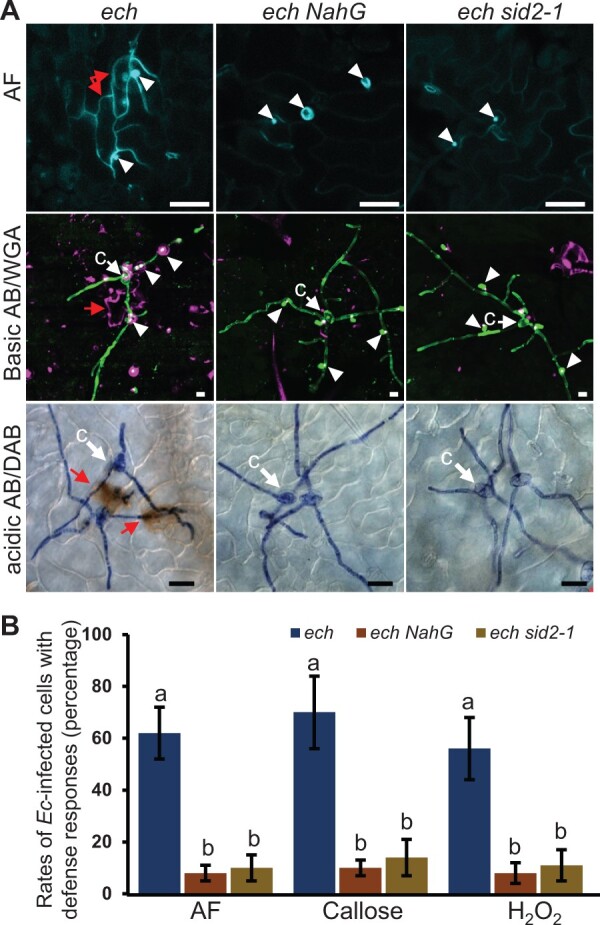
The *Ec* infection-induced HR cell death in the *ech* mutant is SA dependent. A, Leaves of 4-week-old plants of *ech*, *ech NahG*, and *ech sid2-1* were detected for cell death responses including accumulations of lignin-like autofluorescence compounds, callose, and H_2_O_2_ in *Ec*-infected epidermal cells at 3 dpi. Fungal structures on the leaf surface were stained with WGA or acidic AB. The penetration sites were pointed with arrowheads and *Ec*-infected cells with autofluorescence or callose-intense wall or H_2_O_2_ accumulation were indicated with red arrows. Bars = 10 μm. B, Quantitative analysis of *Ec*-infected epidermal cells with autofluorescence, callose-intense wall or H_2_O_2_ accumulation. Mean ± sd, *n* = 5 in which more than 50 penetration sites each were scored. Significant differences were determined by one-way ANOVA with Tukey’s HSD *P* < 0.05.

SA accumulation was reportedly closely related to the HR cell death. To investigate whether the HR cell death induced by *Ec* infection associated with the activation of SA-dependent innate immunity in *ech* plants, we used various tissue-staining techniques to detect the HR cell death responses in the mutants *ech*, *ech NahG*, and *ech sid2-1* during *Ec* infection. The results showed that the intense autofluorescence or aniline-blue stained callosic cell walls present in the *Ec*-infected *ech* cells were nearly eliminated in the *Ec*-infected cells of *ech NahG* and *ech sid2-1* plants (Figure 5, A and B). Furthermore, the H_2_O_2_ accumulation associated with the *Ec*-infected *ech* cells was barely detected in the *ech NahG* and *ech sid2-1* mutants. These results supported that the HR cell death was induced by *Ec* infection in the *ech* mutant and depended on the SA-mediated innate immunity.

On the other hand, the double mutant *ech jar1-1* had no obvious differences from the *ech* mutant in the powdery mildew resistance (Figure 4, B and C). Measurement of JA contents in the leaf tissues revealed that the JA contents were decreased in the *ech* mutant comparing to the WT plants after *Ec* inoculation (Supplemental Figure S2, B and C). Furthermore, a full set of genes involved in JA biosynthesis and signaling were not concertedly regulated in the *ech* mutant before or after *Ec* inoculation (Supplemental Figure S2A; Supplemental Table S5). Collectively, the JA signaling pathway was unlikely associated with the enhanced powdery mildew resistance in the *ech* mutant.

### ECH loss induces SA-independent spontaneous cell death

**Figure 6:**
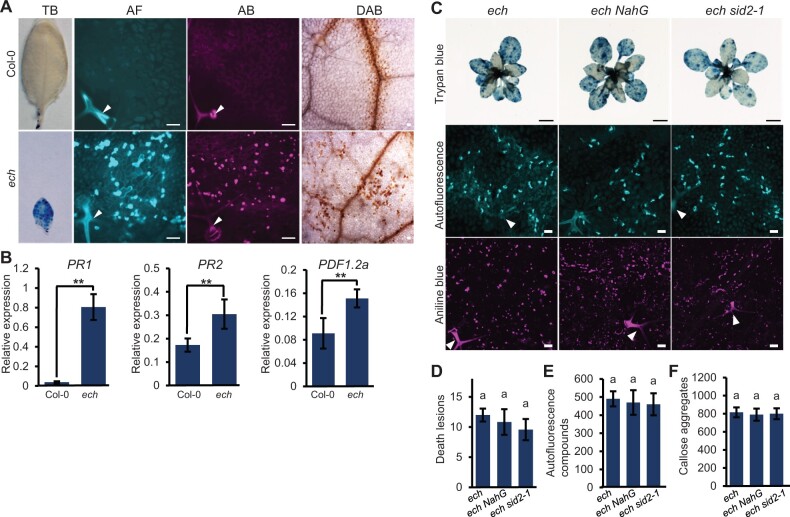
The spontaneous cell death present in the *ech* mutant is SA independent. Leaves of 4-week-old WT and mutant plants grown under normal conditions were examined for the spontaneous cell death and associated responses. A, Detection of the spontaneous cell death lesions and accumulations of lignin-like autofluorescence compounds, callose, and H_2_O_2_ in the WT and *ech* plants by various tissue staining. Bars = 100 μm. B, Analysis of the relative expressions of defensive genes *PR1, PR2*, and *PDF1.2a* by RT–qPCR (means ± sd, *n* = 6, in which six samples were scored). Arabidopsis *UBQ5* was used as the internal control. One-way ANOVA with Tukey’s HSD *P* < 0.05. C, Detection of the accumulations of lignin-like autofluorescence compounds, callose, and spontaneous cell death lesions in the plants of *ech*, *ech NahG*, and *ech sid2-1*. Bars = 1 cm in the first row, and 200 μm in the second and third rows. D, Quantitative determination of the cell death lesions in mature leaves per square centimeters (means ± sd, *n* = 5, in which five leaves each were scored). One-way ANOVA with Tukey’s HSD *P* < 0.05. E and F, measurement of autofluorescence compounds and callose aggregates in the mature leaves per square millimeters, represented as means ± sd (*n* = 10, in which six sites each were scored). Significant differences were determined by one-way ANOVA with Tukey’s HSD, *P* < 0.05. Arrowheads indicate the trichomes in (A) and (C). TB, trypan blue.

As shown in Figure 2A, widespread callose aggregates unassociated with *Bgh* infection were observed in the *ech* leaf tissues, suggesting the presence of extreme stress or even spontaneous cell death in *ech* plants. Spontaneous cell death could be induced by loss-of-function mutations in many key negative regulators in Arabidopsis and occurs in the absence of pathogens (Chakraborty et al., 2018). We performed trypan blue staining, a widely used technique to detect the cell death in plants, to examine whether spontaneous cell death occurred in *ech* leaf tissues. Indeed, massive blue macroscopic death lesions were detected across the *ech* leaf surface whereas only the wounding site of petiole caused by sampling was observed with small blue macroscopic death lesions in the WT plants (Figure 6A). Next, we examined cell death-associated responses, including lignin-like autofluorescence compounds, callose, and H_2_O_2_ accumulations in *ech* under pathogen-free conditions. As expected, the *ech* leaf tissues deposited runaway lignin-like autofluorescence compounds, callose and H_2_O_2_ (Figure 6A). In contrast, these compounds were only deposited in specific leaf tissues, that is, the trichomes or the vascular bundles in the WT (Figure 6A). We tested the expression levels of the defense response marker genes *PR1*, *PR2*, and *PDF1.2a* by RT–qPCR (Reverse Transcription-quantitative Polymerase Chain Reaction), and found they were constitutively upregulated in *ech* compared to the WT (Figure 6B). These findings demonstrated that ECH disruption induced accelerated spontaneous cell death and stress responses in the *ech* mutant.

Next, we wanted to address whether the spontaneous cell death and stress responses were associated with the activation of SA-mediated innate immunity in *ech* plants. First, we performed trypan blue staining of the whole plants of *ech*, *ech NahG*, and *ech sid2-1* mutants to detect the spontaneous cell death. Unexpectedly, the intense blue macroscopic lesions detected in the leaves of *ech* plants were also present in that of *ech NahG* and *ech sid2-1* plants (Figure 6C) and the magnitudes of spontaneous cell death in *ech NahG* and *ech sid2-1* were only slightly reduced compared to that in *ech* revealed by the quantitative analysis of the blue macroscopic death lesions (Figure 6D). Additionally, the stress responses including the ectopic accumulations of lignin-like autofluorescence materials and callose in *ech* were also detected in the leaf tissues of *ech NahG* and *ech sid2-1* (Figure 6C). The magnitudes of stress responses were not significantly alleviated by the introduction of *NahG* or *sid2-1* into the *ech* mutant (Figure 6, E and F). From these results, we concluded that the spontaneous cell death and stress responses in the *ech* mutant were independent of SA-mediated immunity.

### Constitutive callose accumulation induced by ECH disruption is PMR4-dependent

**Figure 7:**
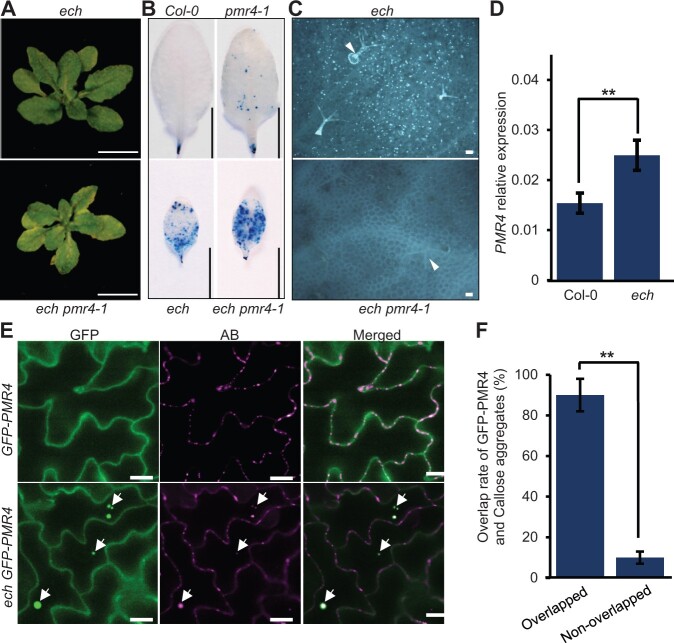
Constitutive callose deposition induced by ECH disruption is PMR4 dependent. A, The phenotypes of 5-week-old *ech* and *ech pmr4-1* plants. Bars = 1 cm. B, Cell death detection in the mature leaves by trypan blue staining. Bars = 1 cm. C, Callose depositions detected in mature leaves of *ech* and *ech pmr4-1* plants. The trichomes are indicated with arrowheads. Bars = 100 μm. D, RT–qPCR analysis of the relative expression of *PMR4* in the WT and *ech* plants (means ± sd, *n* =  3, in which five plants each were scored). Student’s *t* test, ^**^P < 0.01. E, Colocalization analysis of intracellular GFP-PMR4 and callose aggregates in mature leaves of the WT and *ech* plants expressing *pUBQ:GFP-PMR4*. The colocalized sites of GFP-PMR4 and callose aggregates were indicated with arrows in the epidermal cells of *ech* plants. Bars = 10 μm. F, Measurement of the colocalized GFP-PMR4 and callose sites in *ech* mature leaves (means ± sd, *n* = 3, in which more than 100 sites each were scored). Student’s *t* test, ^**^P < 0.01.

The constitutive callose accumulation in the *ech* mutant plants prompted us to investigate the underlying mechanisms. In the genome of *A. thaliana* ecotype Col-0, 12 callose synthases (CalS or GSL for Glucan Synthase-like) have been reported in total and the CalS12 (also known as PMR4) is reportedly responsible for the callose deposition in response to abiotic and biotic stresses (Jacobs et al., 2003; Nishimura et al., 2003). We tested whether PMR4 was associated with the constitutive callose deposition in the *ech* mutant. The ethylmethane sulfonate mutation allele *pmr4-1*, which produces a stop codon in the second exon of *PMR4* gene and results in a nonfunctional truncated PMR4 polypeptide (Nishimura et al., 2003), was introduced into the *ech* mutant. The double mutant *ech pmr4-1* appeared in accelerated dwarf phenotype with obvious chlorosis on the mature leaf margins (Figure 7A). Trypan blue staining revealed the blue death lesions scattered on the mature leaves of the *pmr4-1* mutant and accelerated blue death lesions on the mature leaves of *ech pmr4-1* plants compared to the *ech* plants (Figure 7B). However, aniline blue staining revealed that the abundant callose aggregates detected in the leaves of *ech* plants were completely eliminated in those of *ech pmr4-1* plants (Figure 7C), demonstrating that the constitutive callose deposition induced by ECH loss was PMR4 dependent.

We then investigated how PMR4 is affected by *ECH* loss mutation. We carried out RT–qPCR to determine *PMR4* expressions in the WT and *ech* plants, and found that in contrast to the WT, *PMR4* expression was moderately upregulated in *ech*, about 1.6-folds higher (Figure 7D). To investigate the impact of ECH loss on PMR4 subcellular localization, we crossed the WT plants expressing GFP-PMR4 with the ech plants and introduced the GFP-PMR4 into the *ech* mutant. Confocal microscopy showed that the GFP-PMR4 signal in the WT plants displayed a continuous PM distribution pattern (Figure 7E), consistent with a previous report (Ellinger et al., 2013). In contrast, GFP-PMR4 in the *ech* cells formed intracellular spheroid bodies beside the PM distribution pattern (Figure 7E), reminiscent of the distributions of GFP-PEN1, PEN3-GFP, and the PMA-GFP in the *ech* cells (Figure 1A), indicating a secretion defect of PMR4 in the *ech* mutant. The leaf epidermal cells of WT and *ech* plants expressing GFP-PMR4 were also detected for callose depositions by aniline blue staining (Figure 7E). In the WT cells, only punctate calloses at the plasmodesmata were detected around the cell walls, whereas in the *ech* cells, both the punctate calloses at the plasmodesmata and the intracellular accumulated callose aggregates were detected in the *ech* cells (Figure 7E). Importantly, we found that the intracellular callose aggregates were colocalized with the retained intracellular GFP-PMR4 bodies and the overlapped rates were determined as high as 90% in the leaf epidermal cells of *ech* plants (Figure 7, E and F). Taken together, ECH loss-mediated secretion defect resulted in a relocalization of PMR4 which may be associated with the induction of PMR4 enzymatic activity.

### ECH disruption induces chronically prolonged ER stress

**Figure 8:**
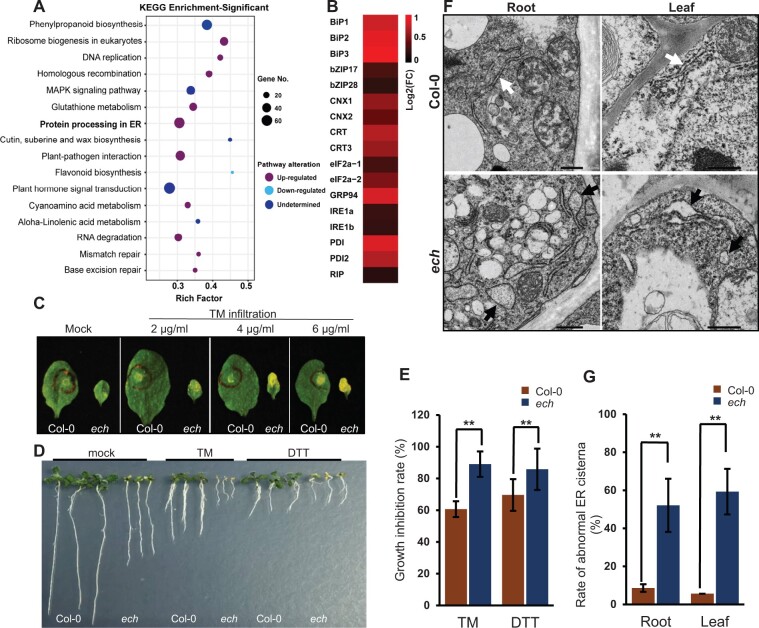
ECH disruption induces a chronically prolonged ER stress. A, The pathway PPIER was significantly enriched and upregulated in the *ech* mutant based on KEGG enrichment of DEGs from a comparison between the RNAseq data of WT and *ech* plants without *Ec* inoculations. The significantly enriched biological pathways were displayed (adjusted *P* < 0.05). B, Expression profiles of ER stress-associated genes. C, Sensitivity test of the WT and *ech* plants to TM. Representative mature leaves were photographed at 3 days after TM infiltration. D, Sensitivity test of the WT and *ech* seedlings to TM or DTT. Representative seedlings were photographed at 8 days after TM or DTT addition into the MS medium. Notably, severe chlorosis symptoms were present in the leaves of *ech* seedlings but not the WT seedlings after treatment with TM or DTT. E, Quantitative determination of inhibitions of the root growth after treatment with TM or DTT (means ± sd, *n* = 5, in which >20 seedlings each were scored). Student’s *t* test, ^**^P < 0.01. F, TEM examination of ER morphology in the root or leaf cells of the WT and *ech* plants. The representative normal and dilated ER tubules are indicated with white and black arrows, respectively. Bars = 500 nm. G, Measurement of dilated ER tubules in the root and leaf cells of the WT or *ech* plants (means ± sd, *n* = 3, in which more than 100 ER tubules each were scored). Student’s *t* test, ^**^P < 0.01.

We used the RNAseq data to profile the effects of ECH disruption on the biological pathways on a genome-wide scale. In total, 6,357 differentially expressed genes (DEGs) were identified between the RNAseq data of *ech* and WT plants without *Ec* inoculations (Mock, 2 dpi, Supplemental Table S2), which were used for the Kyoto Encyclopedia of Genes and Genomes (KEGG) enrichment. The results showed that 16 biological systems were significantly enriched most of which were found to be upregulated (Figure 8A; Supplemental Table S6). Among them, the upregulation of the biological pathway PROTEIN PROCESSING IN ER (PPIER) implies a striking effect of *ECH* loss on the ER functions (Figure 8A; Supplemental Figure S3 and Supplemental Table S6). Further analysis showed that most ER chaperons such as *CRT*, *CNX*, *Bip2*, *Bip3*, *GRP94*, *PDI*, etc., which are critical for protein folding, processing, and modifications in ER (Wan and Jiang, 2016), were coordinately upregulated in the *ech* mutant compared to the WT plants (Figure 8B; Supplemental Tables S6 and S7), suggesting an extreme ER stress that the *ech* mutant underwent. To confirm that, we tested the WT and *ech* plants for sensitivity to tunicamycin (TM) or dithiothreitol (DTT), which can induce ER stress by inhibiting protein glycosylation or disulfide bond formation (Neubauer and Innes, 2020). Injection of rosette leaves with TM solution triggered chlorosis and necrosis of the injected leaf region much faster in *ech* plants than in the WT plants (Figure 8C). After addition of TM into the Murashige and Skoog (MS) medium, the root growth of *ech* seedlings was much slower than that of the WT seedlings (Figure 8, D and E). Furthermore, TM treatment induced an accelerated chlorosis symptom in the leaves of *ech* seedlings but not in that of WT seedlings (Figure 8D). DTT treatment also showed similar effects as that of TM treatment on the WT and *ech* seedlings (Figure 8D). Chronic ER stress always results in alteration and malformation in ER morphology in cells (Wan and Jiang, 2016). We then performed transmission electron microscopy (TEM) to examine the ER morphology in the mature leaf and root cells of *ech* and WT plants grown under normal conditions. In the WT plants, long consistent ER tubules were observed frequently confining to the cytoplasm periphery of root or leaf cell (Figure 8F). In contrast, most of the ER tubules in *ech* displayed altered morphology and dilations in both leaf and root cells (Figure 8, F and G). Particularly, some of the abnormal ER tubules in *ech* cells dilated to fusiform bodies (Figure 8F), which, however, were not observed in WT cells. These results supported that ECH was required for maintaining the normal functions of ER and *ECH* loss induced a chronically prolonged ER stress.

## Discussion

Plant immunity is tightly and spatio-temporally regulated by various mechanisms and executes at different levels and appropriate magnitudes to avoid damaging of the plant growth and development. In this study, we found that the TGN component protein ECH plays important roles in multilayered regulations of plant immunity as well as stress responses.

Plant penetration resistance is established by site-directed deposition of various materials at the pathogen-infected sites to constitute a physical and chemical barrier (Underwood and Somerville, 2008; Meyer et al., 2009). Our study together with previous studies reveals the essential role of ECH-mediated TGN secretion for the penetration resistance by maintaining various cargoes to the cell surface, including the PM proteins GFP-PEN1, PEN3-GFP (Figure 1) and VAMP 721 (Uemura et al., 2019), the extracellular antimicrobial proteins PDF1.2a-GFP and PR1-GFP (Figure 1), and the cell wall polysaccharides pectin and hemicellulose, and cell wall-modified enzymes (Gendre et al., 2013; Uemura et al., 2019). Despite compromising the penetration resistance, our study found that ECH loss resulted in unexpected enhanced powdery mildew resistance (Figure 3). We demonstrated that this resistance mainly resulted from the constitutive activation of SA-mediated innate immunity due to ICS1-derived SA hyperaccumulation in the *ech* mutant, although ER stress may also contribute to the enhanced postinvasion resistance (Figure 4). How *ECH* loss induces *ICS1* upregulation and enzymatic activation to produce SA remains to be determined. Given the different subcellular localizations of ECH (in the TGN) and ICS1 (in the chloroplast), the regulation of *ICS1* expression by *ECH* is unlikely direct. Notably, SA hyperaccumulation was also reported in the double mutant *syp42 syp43* of which the secretory capacity of CSP was defective (Uemura et al., 2012), indicating a potential intrinsic link between the CSP pathway and SA biosynthesis regulation.

Previous studies reported that the SA signaling plays a dual role in the regulation of cell death in plants whereas the exact mechanism is unclear (Radojičić et al., 2018). Our study revealed two different types of cell death in the *ech* mutant, that is, the SA-dependent HR cell death during *Ec* infection and the SA-independent spontaneous cell death (Figures 5 and 6), further confirming the complicated regulations of cell death in plants. In the Arabidopsis double mutant *syp42 syp43* with a secretion defect, SA hyperaccumulation and the spontaneous cell death are also reported whereas they are coupled in *syp42 syp43* (Uemura et al., 2012). There may be an intrinsic link between the CSP and the regulation of SA biosynthesis and cell death. However, the underlying mechanisms are largely unknown. Notably, accumulations of intracellular aggregates were reported to induce autolytic programmed cell death in the hybrid cells of different *Nicotiana* species (Ueno et al., 2019). Given the abundant aggregates of various proteins formed inside *ech* cells, autolytic programmed cell death may be triggered and responsible for the spontaneous cell death in the *ech* mutant. On the other hand, the spontaneous cell death may be a direct consequence of ER stress, because ER stress-induced cell death has been well studied in plants (Wan and Jiang, 2016). The spontaneous cell death is likely responsible for the increased susceptibility of *ech* plants to the necrotrophic pathogen *S. sclerotiorum*, considering its nutrient-uptaking strategy from the dead plant tissues.

Among the stress responses, constitutive callose depositions were observed in the *ech* mutant and depended on the PMR4 enzymatic activity (Figure 7). Our study revealed that *PMR4* had quite an amount of basal expression level in the WT plants (Figure 7D), whereas no PMR4-derived callose aggregates were detected in the leaf tissues (Figure 6A). Particularly, when *PMR4* expression was mandatorily driven by a constitutive promoter in the WT plants, no ectopic callose deposition was detected in the leaves of plants grown in normal conditions in our study (Figure 7E) and previous studies (Ellinger et al., 2014; Kulich et al., 2018). This indicates that the enzymic activity of PMR4 to produce callose is likely posttranscriptionally regulated. Our results showed that the translocation of PMR4 may be associated with the activation of PMR4 enzymic activity supported by the colocalizations of the intracellular PMR4 and callose aggregates in *ech* cells (Figure 7, E and F). Induction of callose synthase activity of NaGSL1 by its relocalization was reported in *Nicotiana alata* during the transition of different development stages (Brownfield et al., 2008). Therefore, the translational modification and translocation may be key for the activation of PMR4 callose synthase activity.

It has been reported that ER stress could be induced by mutations in ER-localized proteins, for example, the ER chaperons Bip2 and bZIP17/28, and the acetyltransferase NAA50 (Wang et al., 2005; Liu et al., 2007; Neubauer and Innes, 2020). In our study, loss of the TGN component protein ECH also induced severe ER stress (Figure 8). This report shows that disruption of a TGN protein could induce ER stress in eukaryotic cells, suggesting an intrinsic link between the TGN and ER. Previous study reported that the secGFP aggregates retained inside the *ech* cells were partially colocalized with the ER marker BIP (Gendre et al., 2011), implying that ECH loss-mediated secretion defect could lead to protein accumulations in ER which overwhelm the ER processing capacity and could result in ER stress in eukaryotic cells (Wan and Jiang, 2016). Additionally, different from the mammalian cells, the ER and Golgi complex in plant cells are physically contacted at the ER exit sites (Liu and Li, 2019) and hence have more closed interactions. Therefore, the secretion defect of the TGN may result in protein overaccumulations in ER and induce ER stress.

**Figure 9:**
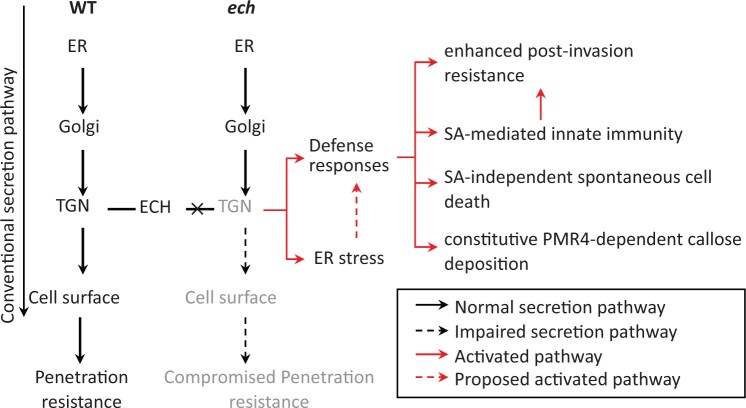
A diagram illustrating the roles of ECH in multilayered regulation of plant resistance and stress responses. In the cells of WT plants, ECH acts as an essential secretion factor for moving various proteins to the cell surface and thereby contributes to the penetration resistance. ECH loss induces ER stress and defense responses including enhanced postinvasion resistance, SA-mediated innate immunity, SA-independent spontaneous cell death and constitutive PMR4-dependent callose deposition. In the *ech* mutant, SA-mediated innate immunity is largely responsible for the enhanced postinvasion resistance, and ER stress may be associated with the activation of these defense responses.

Finally, we propose a model to illustrate the roles of ECH in multilayered regulations of plant resistance and stress responses (Figure 9). In the WT plants, the TGN component ECH maintains the full secretory capacity of CSP to deliver various defense cargoes from the TGN to the cell surface, and thereby contributes to the penetration resistance. ECH disruption results in ER stress and activation of defense responses including the enhanced postinvasion resistance, the SA-mediated innate immunity, the SA-independent spontaneous cell death, and the constitutive PMR4-dependent callose deposition. In the *ech* mutant, the ER stress may be associated with the activation of these defense responses and among them, the SA-mediated innate immunity is largely responsible for the enhanced postinvasion resistance.

## Materials and methods

### Plant materials and growth

All the Arabidopsis (*A. thaliana*) plants were grown in soil (Sunshine #1 N&O Mix, Sun Gro Horticulture Canada Ltd., Canada) under normal conditions with 21°C and a 16-h photoperiod of ∼125 μE m^−2^s^−1^. The Arabidopsis mutants used in this study were obtained from the ABRC stock center (Col-0 background), including *ech* (SAIL_163_E09) (Gendre et al., 2011), *sid2-1* (Nawrath and Métraux, 1999), *jar1-1* (Staswick et al., 2002), *ein2-1* (Roman et al., 1995), *pmr4-1* (Nishimura et al., 2003), and the transgenic line *NahG* (Lawton et al., 1995). The Arabidopsis transgenic lines tagged with fluorescent proteins include: *pECH:ECH-YFP* (Gendre et al., 2011), *pPEN1:GFP-PEN1* (Collins et al., 2003), *pPEN3:PEN3-GFP* (Stein et al., 2006), *35S:PDF1.2a-GFP* (Watanabe et al., 2013), *35S:PR1-GFP* (Watanabe et al., 2013), *35S:secRFP* (Faso et al., 2009), *35S:PMA-GFP* (Lefebvre et al., 2004), and *pUBQ:GFP-PMR4* (Kulich et al., 2018). The homozygosity of T-DNA insertion mutants was genotyped by PCR with both gene-specific and T-DNA border primers. The point mutation lines were genotyped by sequencing the PCR products after amplification with gene-specific primers (Thermo Fisher Scientific, Canada). The primers used in this study are listed in Supplemental Table S1.

### Pathogen inoculation

The cucumber powdery mildew fungus *Ec* and the barley powdery mildew fungus *Bgh* were maintained and propagated on the plants of cucumber (*Cucumis sativus*, variety Sweet Slice) and barley (*Hordeum vulgare* L, cultivar CDC silky), respectively. Four-week-old Arabidopsis plants were inoculated with conidiospores of *Ec* or *Bgh* at the density of 5–10 conidia mm^−2^. For inoculation, 12 Arabidopsis plants (3 pots) of each indicated line were placed in settling towers and inoculated with conidia by heavily tapping the infected cucumber or barley leaves above the Arabidopsis plants. One or two leaves of each inoculated plant were sampled and examined under a microscope to determine the conidial numbers and 10 sites were scored for calculation of the conidial density on the leaves.

### Microscopy and imaging

Fresh inoculated or noninoculated leaves of Arabidopsis plants were used to detect the autofluorescence compound accumulation under the ultraviolet excitation with a confocal laser scanning microscope (ZEISS LSM 880). Fixed inoculated or noninoculated leaves were stained with alkaline aniline blue solution (pH 9.6, 2 mg mL^−1^) for callose, acidic aniline blue solution (pH 5.0, 2 mg mL^−1^) for fungal structures or 3,3′-diaminobenzidine solution (DAB, 2 mg mL^−1^) for H_2_O_2_ accumulation as described previously (Liu et al., 2010). Macroscopic detection of cell death on the leaves was monitored by lactophenol-trypan blue staining (Koch and Slusarenko, 1990).

Fresh leaves were incubated in 10-μM propidium iodide (PI, Life Technologies, Canada) or 10-μM DAPI (Sigma-Aldrich, Canada) solutions for 10 min followed by confocal microscopy to visualize the cell wall. For plasmolysis, fresh leaves were placed on a slide, soaked in 0.8-M KCl solution and mounted with a cover glass followed by confocal microscopy. The fluorescence was detected under a confocal microscope with the following setting of excitation/emission wavelengths: autofluorescence compound, callose and DAPI (405/420–480 nm), WGA (WGA) or GFP (488/505–530 nm), RFP (543/581–635 nm), and PI (488/>560 nm).

### Hormone quantification

Four-week-old Arabidopsis plants with or without *Ec* inoculation were used for SA and JA quantification. The rosette leaves of plants were harvested at 2 dpi and immediately frozen with liquid nitrogen for further analysis of SA and JA, and three biological replicates were carried out for each treatment. In each biological replicate, the rosette leaves of 15 WT or *ech* plants with or without *Ec* inoculation were collected and subjected to hormone quantification. The extraction and quantification of SA and JA were performed at the Aquatic and Crop Resource Development Research Center, NRC (Saskatoon, Canada) as described previously (Murmu et al., 2014). Briefly, the frozen plant tissues were ground and extracted with methanol–water–glacial acetic acid solutions (90:9:1, v/v/v) followed by successive incubation, centrifugation, purification, and condensing to prepare SA and JA solutions. Quantification analysis was performed by ultra-performance liquid chromatography–electrospraytandem mass spectrometry (UPLC/ESI–MS/MS) using a Waters ACQUITYUPLC system (Waters Limited, Canada). MassLynx and QuanLynx (Micromass, UK) were used for data acquisition and analyses.

### Transcriptome profiling

Four-week-old Arabidopsis plants with or without *Ec* inoculation were used for transcriptome sequencing. The rosette leaves of mock and *Ec*-inoculated plants were harvested at 2, 5, and 7 dpi, respectively, and immediately frozen with liquid nitrogen for total RNA extraction. Three biological replicates were set up for each treatment. In each biological replicate, the rosette leaves of five WT or *ech* plants with or without *Ec* inoculation at each time point were collected and subjected to transcriptome sequencing. Total RNA was extracted from ∼0.1 g of each sample (fresh weight) using the Plant RNA Isolation Mini Kit (Agilent Technologies, Canada) according to the user manual. cDNA library construction and sequencing, data quality control and gene expression calculation were performed in BGI Genomics Co., Limited (Shenzhen, China). A comparison between the RNAseq data of WT and *ech* plants without *Ec* inoculation were performed to identify the DEGs with the thresholds of FPKM (Fragments per kilobase per million mapped fragments) value >1, |log2(gene foldchange, FC)| ≥ 1 and adjusted *P* < 0.05. KEGG enrichment and heatmap analysis of the resulting DEGs were carried out with the OmicShare toolkit (http://www.omicshare.com/tools).

### RT–qPCR

RT–qPCR analysis was carried out with the cDNA synthesized from total RNA isolated as above methods. Gene-specific primers were listed in Supplemental Table S1. The Arabidopsis gene *UBIQUITIN 5* (*UBQ5*, AT3G62250) was used as the internal control. The relative expressions of target genes were calculated using the resultant C_t_ values and tested with a two-tailed Student *t* test for significant differences.

### Chemical treatments

Two chemical agents TM (Apexbio) and DTT (Roche) were used to test the Arabidopsis seedlings or adult plants for sensitivity to ER stress. TM and DTT were prepared for 1.0-mg mL^−1^ stock solutions in DMSO (Dimethyl sulfoxide) and 100-mg mL^−1^ stock solutions in ultrapure water, respectively. For Arabidopsis seedling sensitivity test, seeds were sterilized with 70% (v/v) ethanol solution and mounted on 1/2 MS medium supplemented with TM (0.2 μg mL^−1^) or DTT (100 μg mL^−1^) after washing the seeds with sterile water. The seedlings grown in plant growth chamber were investigated after 8 days treatment. For adult plant sensitivity test, leaves of 5-week-old plants were respectively injected with a series of gradient TM solutions (2.0 μg mL^−1^, 4.0 μg mL^−1^, and 6.0 μg mL^−1^) with a needleless syringe and the result was harvested 3-day posttreatment when severe leaf chlorosis appeared in the *ech* plants.

### TEM

For TEM analysis, the roots of 8-day-old-seedlings of Arabidopsis grown on 1/2 MS medium and the young leaves of 4-week-old plants grown in plant growth chamber were sampled for tissue-embedded block preparation, ultrasection and sample staining as described previously (Liu et al., 2020). TEM observation was performed with a transmission electron microscope (JEM-1400 Flash, Japan).

### Statistical analysis

The significant differences were calculated with a two-tailed Student t test for paired comparison or one-way ANOVA (Analysis of variance) with Tukey’s honestly significant difference for multiple comparisons. Groups or samples with statistically significant differences are marked with asterisk(s) (^*^P <  0.05; ^**^P < 0.01, Student *t* test) or different letters (*P* < 0.05, lowercase letters, ANOVA). Error bars represent standard deviations based on at least three replicates.

### Data availability

The RNA-seq data generated from this study are available under the accession number PRJNA663433 from the National Center for Biotechnology Information Gene Expression Omnibus (NCBI-GEO) database. The general information of the RNA-seq data was listed in Supplemental Tables S2 and S3.

### Accession numbers

Sequence data from this article can be found in the GenBank/EMBL data libraries under accession numbers: AT1G09330 (ECH), AT2G14610 (PR1), AT5G44420 (PDF1.2a), AT3G11820 (PEN1), AT1G59870 (PEN3), AT2G18960 (PMA or AHA1), AT1G74710 (SID2 or ICS1), AT2G46370 (JAR1), AT5G03280 (EIN2), and AT4G03550 (PMR4).

## Supplemental data

The following materials are available in the online version of this article.

**Supplemental Figure S1.** Resistance test to the necrotrophic pathogen *S. sclerotiorum.*

**Supplemental Figure S2.** Effects of *ECH* loss on the gene expressions of JA pathway.

**Supplemental Figure S3.** Gene expression of the PPIER pathway altered by *ECH* loss.

**Supplemental Table S1.** PCR primers used in this study.

**Supplemental Table S2.** General information of the RNA-seq data.

**Supplemental Table S3.** Correlations between replicates based on RNA-seq data.

**Supplemental Table S4.** The expression profiles of genes involved in SA signaling pathway before and after *Ec* inoculations.

**Supplemental Table S5.** The expression profiles of genes involved in JA signaling pathway before and after *Ec* inoculations.

**Supplemental Table S6.** The significantly affected biological processes by ECH disruption revealed by KEGG enrichment analysis and the DEGs of each biological process.

**Supplemental Table S7.** The expressions of ER stress indicative genes altered by ECH loss.

## Supplementary Material

kiac400_Supplementary_Data

## Notes

L.L. and Y.W. initiated the project. L.L., L.Q., and Y.W. performed microscopic analyses. C.Z., F.G., and X.C. generated double mutants and conducted pathogenicity tests. L.B.S., Z.B., M.X., and Y.Z. conducted gene expression profiling, RT-qPCR, and analysis of plant hormone measurement. J.H. performed the TEM analysis. L.L., L.Q., and Y.W. wrote the article with input from L.B.S., S.L., and R.P.B.

The authors responsible for distribution of materials integral to the findings presented in this article in accordance with the policy described in the Instructions for Authors (https://academic.oup.com/plphys/pages/general-instructions) are: Yangdou Wei (yangdou.wei@usask.ca) and Shengyi Liu (liusy@oilcrops.cn).
