# Supplementary material for: The plant trans-Golgi network component ECHIDNA regulates defense, cell death, and endoplasmic reticulum stress
Source: Plant Physiol. 2022 Aug 26;191(1):558–74. doi: 10.1093/plphys/kiac400 (PMC9806577; doi:10.1093/plphys/kiac400)
Supplement: kiac400_Supplementary_Data [file kiac400_supplementary_data.zip › kiac400_Supplementary_Data/PP2022RA00972D_Supplemental_Table_1.pdf]

Supplemental Table 1. PCR primers used in this study.

Gene name	Gene ID	Primer name	Primer sequence	PCR product size (bp)
UBQ5	AT3G62250	UBQ5-F	GACCAGCAGCGATTGATTTTC	124
		UBQ5-R	TCTTCTTAGCACCACCACGGA	
ECH	AT1G09330	ECH-F	TGGTGGAACGAGATCAATGACT	197
		ECH-R	CGCCAACAACAAGCAGATAGTC	
PR1	AT2G14610	PR1-F	CTCGAAAGCTCAAGATAGCCCAC	131
		PR1-R	CTTAGTTGTTCTGCGTACGTCC	
PR2	AT3G57260	PR2-F	CAAGGAGCTTAGCCTCACCACC	130
		PR2-R	GATGGACTTGGCAAGGTATCG	
PDF1.2a	AT5G44420	PDF1.2a-F	TTGCTTCCATCATCACCCTTATCTT	170
		PDF1.2a-R	GGCTCCTTCAAGGTTAATGCAC	
PMR4	At4g03550	qPMR4-F	CTCTTAAAACCGTTGGAGACCTTC	140
		qPMR4-R	ATGAAGCACCATATGCTCACGC	
NahG	-	NahG-F1	GCCTTAGCACTGGAACTCTG	386
		NahG-R1	TCGGTGAACAGCACTTGCAC	
sid2-1	AT1G74710	SID2-F2	CGAGTTCTCTATCGTACGAG	763
		SID2-R	TAGATCAATGCCCCAAGACC	
jar1-1	At2g46370	JAR-F	GGAAACGCTACTGACCCTGA	629
		JAR-R	TCGGGACTACAGGAAGGAGA	
ein2-1	AT5G03280	EIN2-F	TGGAACATGGATGCTCAAAA	533
		EIN2-R	CTTAAGCTGCGGAATGAAGG	
pmr4-1	At4g03550	PMR4-F	AGATCAGGGACATGGGACAG	799
		PMR4-R	TTACCAGCCCAACCAATTTC	

Note: -, non-*Arabidopsis* gene; NA, not applicable
