# Supplementary material for: The plant trans-Golgi network component ECHIDNA regulates defense, cell death, and endoplasmic reticulum stress
Source: Plant Physiol. 2022 Aug 26;191(1):558–74. doi: 10.1093/plphys/kiac400 (PMC9806577; doi:10.1093/plphys/kiac400)
Supplement: kiac400_Supplementary_Data [file kiac400_supplementary_data.zip › kiac400_Supplementary_Data/Supplemental Figures.pdf]

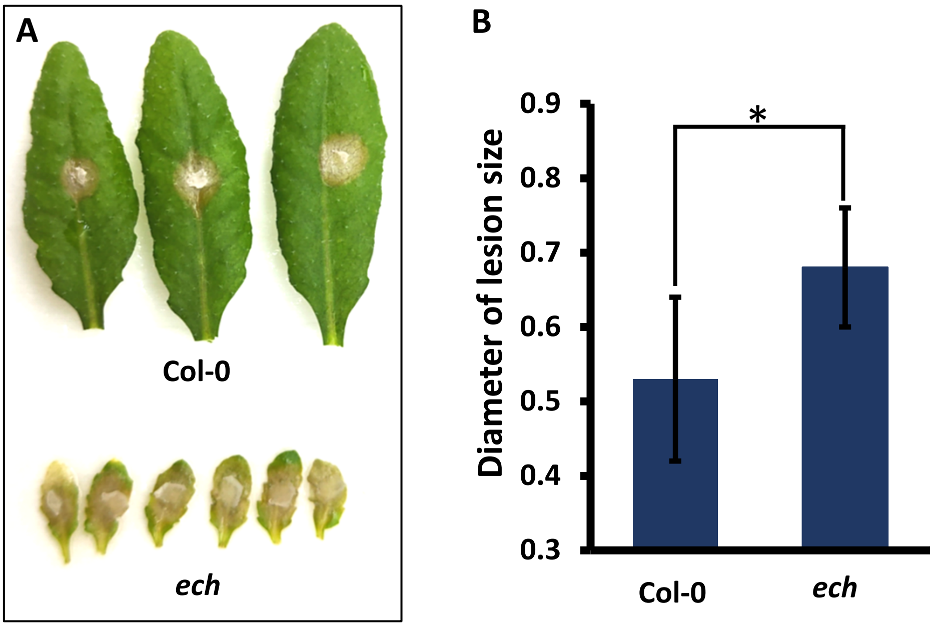

Supplemental Figure S1. Influence of ECH loss on the resistance to *Sclerotinia sclerotiorum.*
Leaves of five-week-old wild-type and mutant plants were inoculated with mycelial plugs of *S. sclerotiorum.* A, The death lesions of S. *sclerotiorum*-inoculated mature leaves at 30 hpi. B, Statistical analysis of the lesion sizes between the wild-type and *ech* plants. The lesion size was measured at 30 hours post inoculation and the means of lesion sizes were presented (n=22 , in which 22 inoculated leaves were scored). Student's *t*-test, *, $p<0.01$.

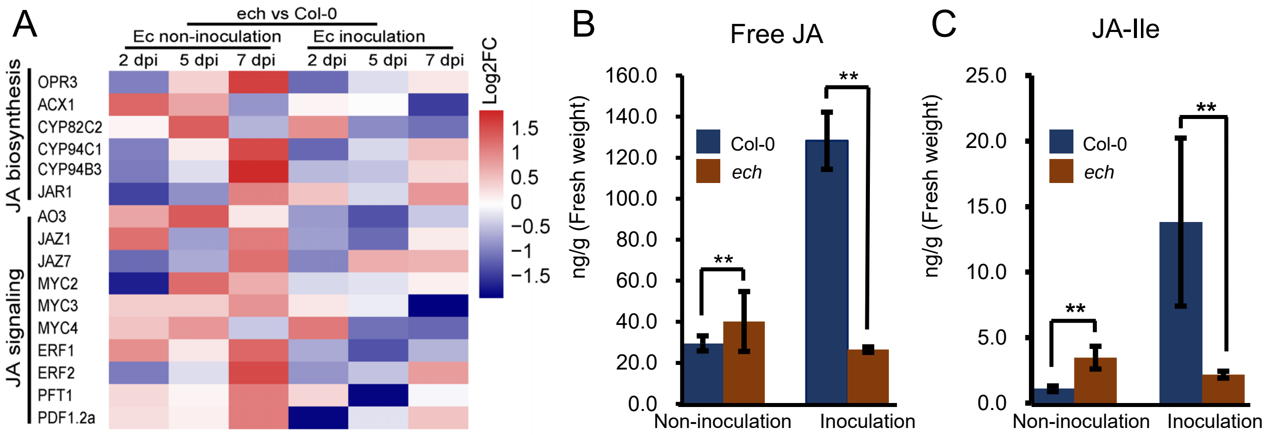

Supplemental Figure S2. Influence of ECH loss on the expressions of jasmonic acid (JA)-associated genes.

A, Expression profiles of the genes involved in JA biosynthesis and signaling pathway without or with *Ec* inoculation at each indicated time point. dpi, days post inoculation. **B** and **C,** Measurement of the free or isoleucine (Ile) conjugated JA content in the leaf tissue of *ech* and Col-0 plants without or with *Ec* inoculation. Mean ± SD, n = 3 in which 15 plants each were performed for JA measurement. Student's *t*-test, **, p < 0.01.

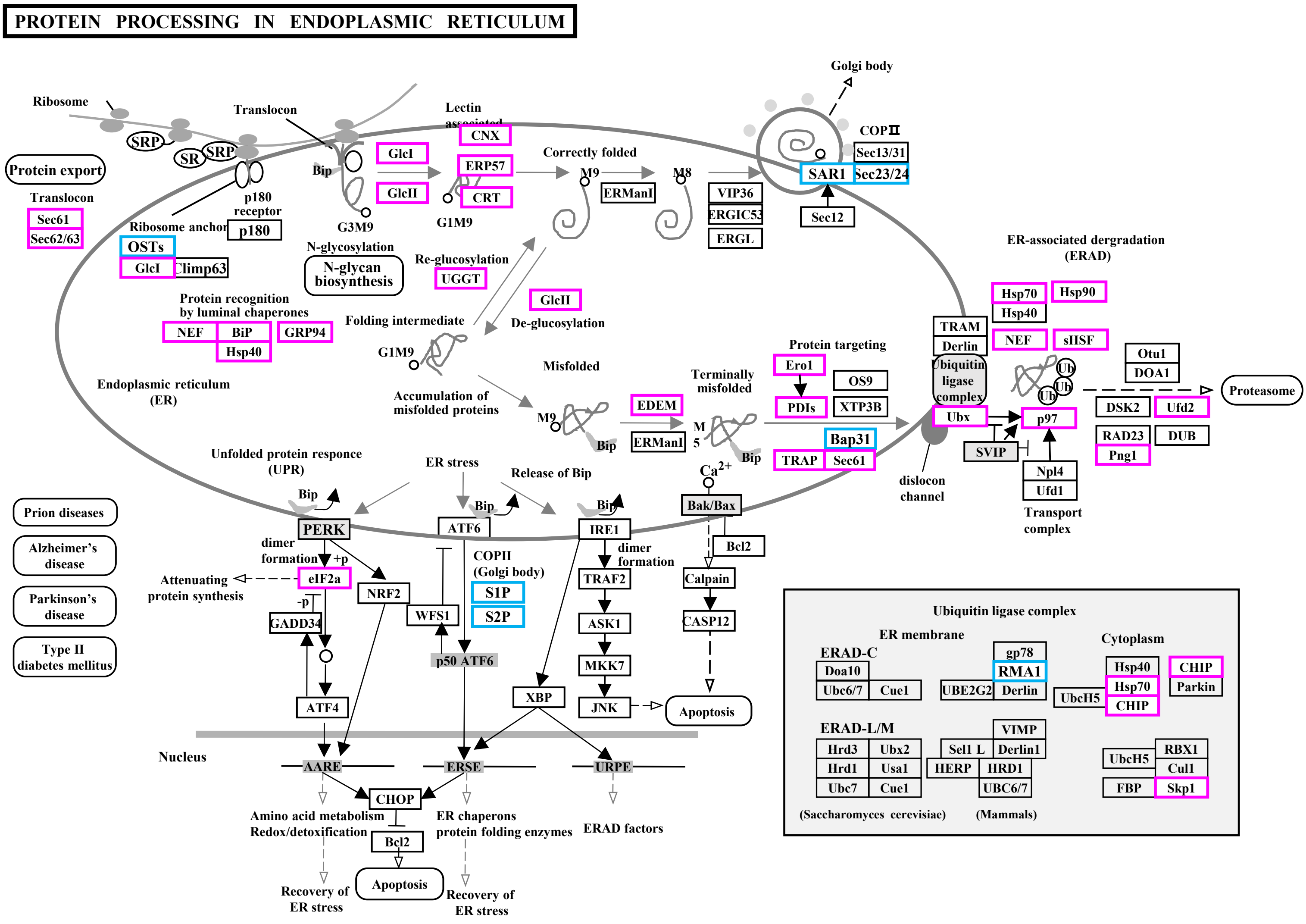

Supplemental Figure S3. Gene expression of the PROTEIN PROCESSING IN ENDOPLASMIC RETICULUM pathway altered by ECH loss.

The gene network model of PROTEIN PROCESSING IN ENDOPLASMIC RETICULUM pathway was downloaded from the KEGG database and further modified for higher resolution. The genes highlighted with purple boxes were upregulated whereas those highlighted with light blue boxes were downregulated in *ech* compared to wild type. The genes highlighted with gray boxes were not massively altered by ECH loss.

Supplemental Table S1. PCR primers used in this study.

Gene name	Gene ID	Primer name	Primer sequence	PCR product size (bp)
UBQ5	AT3G62250	UBQ5-F	GACCAGCAGCGATTGATTTTC	124
		UBQ5-R	TCTTCTTAGCACCACCACGGA	
ECH	AT1G09330	ECH-F	TGGTGGAACGAGATCAATGACT	197
		ECH-R	CGCCAACAACAAGCAGATAGTC	
PR1	AT2G14610	PR1-F	CTCGAAAGCTCAAGATAGCCCAC	131
		PR1-R	CTTAGTTGTTCTGCGTACGTCC	
PR2	AT3G57260	PR2-F	CAAGGAGCTTAGCCTCACCACC	130
		PR2-R	GATGGACTTGGCAAGGTATCG	
PDF1.2a	AT5G44420	PDF1.2a-F	TTGCTTCCATCATCACCCTTATCTT	170
		PDF1.2a-R	GGCTCCTTCAAGGTTAATGCAC	
PMR4	At4g03550	qPMR4-F	CTCTTAAAACCGTTGGAGACCTTC	140
		qPMR4-R	ATGAAGCACCATATGCTCACGC	
NahG	-	NahG-F1	GCCTTAGCACTGGAACTCTG	386
		NahG-R1	TCGGTGAACAGCACTTGCAC	
sid2-1	AT1G74710	SID2-F2	CGAGTTCTCTATCGTACGAG	763
		SID2-R	TAGATCAATGCCCCAAGACC	
jar1-1	At2g46370	JAR-F	GGAAACGCTACTGACCCTGA	629
		JAR-R	TCGGGACTACAGGAAGGAGA	
ein2-1	AT5G03280	EIN2-F	TGGAACATGGATGCTCAAAA	533
		EIN2-R	CTTAAGCTGCGGAATGAAGG	
pmr4-1	At4g03550	PMR4-F	AGATCAGGGACATGGGACAG	799
		PMR4-R	TTACCAGCCCAACCAATTTC	

Note: -, non-*Arabidopsis* gene; NA, not applicable
